# Edible Insects from the Perspective of Sustainability—A Review of the Hazards and Benefits

**DOI:** 10.3390/foods14081382

**Published:** 2025-04-17

**Authors:** Filip Kłobukowski, Maria Śmiechowska, Magdalena Skotnicka

**Affiliations:** 1 Division of Food Commodity Science, Faculty of Health Sciences with the Institute of Maritime and Tropical Medicine, Medical University of Gdańsk, 80-210 Gdańsk, Poland; filip.klobukowski@gumed.edu.pl; 2 Department of Quality Management, Faculty of Management and Quality Science, Gdynia Maritime University, 81-225 Gdynia, Poland; m.smiechowska@wznj.umg.edu.pl

**Keywords:** edible insects, sustainability, nutritional value, 2050, essential amino acid comparison, protein characteristics

## Abstract

The increasing global population, projected to exceed 9.1 billion by 2050, presents a critical challenge for sustainable food production. Edible insects have emerged as a promising alternative protein source due to their high nutritional value, low environmental footprint, and efficient resource utilization. This review explores the opportunities and challenges of integrating edible insects into food systems. Benefits include their high protein content and quality, low greenhouse gas emissions, low-cost production, and ability to thrive on organic waste. Furthermore, edible insect cultivation requires significantly less land and water compared to traditional livestock. Edible insects are nutritionally rich, containing substantial amounts of essential amino acids, unsaturated fatty acids, and minerals. However, barriers to widespread adoption persist, such as cultural perceptions, regulatory hurdles, potential allergenicity, and biological and chemical contamination. Furthermore, standardizing rearing practices and ensuring food safety are critical for broader adoption. While edible insects represent a nutritious, low-cost food and feed, there are a lot of variables that have not been fully investigated. Only after further research, promising results, and solutions that are relatively easy to apply might edible insects be considered a sustainable food source. Considering the challenges that may arise by 2050, more intensive research is highly advised.

## 1. Introduction

According to a report by the FAO, it is anticipated that the human population will continue to grow steadily to over 9.1 billion by 2050 [1], although some sources predict that the population will reach almost 11 billion people [2]. This will pose a tremendous challenge for every aspect of human life. However, the critical issue will be the ability to feed the global population and provide sufficient amounts of drinking water [3,4]. Therefore, in recent years, increased attention has been given to production methods that align with sustainable development principles. To address the challenge of feeding the projected global population by 2050, various strategies have been explored, including the development of salt-tolerant cereals, improvements in fertilizing methods and nanofertilizers, and initiatives such as the creation of super-cereal, which focuses on blending cereals to obtain a more nutritious product [5,6].

The production of sufficient quantities of food with adequate nutritional value while maintaining safety standards throughout the products’ life cycles may prove to be exceptionally difficult. Severe hunger problems have been a worldwide challenge for centuries, especially in developing countries, which already constitute the majority of the human population. The discrepancy in population growth between developing and developed countries is expected to grow intensively [2,7]. Consequently, the hunger problem may increase, further intensifying the need for increased food production. It is crucial to remember that food production, along with other aspects of human living, cannot proceed at the expense of the environment. Therefore, human environmental impacts have been continuously studied for years. It is considered that livestock production is one of the main sources of greenhouse gas emissions [8]. The need to feed and breed livestock might also be a major factor responsible for deforestation and the extremely high demand for water consumption [9]. Considering the above, the search for an alternative, sustainable food source is absolutely crucial to humankind. In order to reduce livestock production, an alternative source of high-quality protein is required. This is the reason for the increase in interest in edible insects [10].

## 2. Opportunities Related to the Popularization, Increased Production, and Consumption of Edible Insects

### 2.1. Sustainable Development

Sustainable development refers to the principles defined by the United Nations Commission in 1987. It is based on the concept of intergenerational equity: “meeting the needs of the present without compromising the ability of future generations to meet their own needs”. It should be noted, however, that while the principle itself is valid, it does not specify ways with which to achieve the goals of the current generations without diminishing the opportunities of future ones. Securing renewability for subsequent generations may significantly hinder the fulfillment of the present generation’s goals. This raises a legitimate question: is intergenerational equity truly achievable?

The United Nations and other organizations are continuously seeking solutions, which in 2015 led to the adoption of 17 Sustainable Development Goals (SDGs). The 2030 Agenda focuses on impressive targets, ranging from eliminating poverty and hunger to achieving health, well-being, peace, and many more [11]. These are all very ambitious goals and will be extremely difficult to achieve. It will require a lot of effort and willingness from all the parties involved and might be even harder to achieve now than it was over a decade ago. Therefore, it is essential that necessary actions are undertaken as soon as possible.

#### Edible Insects Within the Framework of Sustainable Development

The production of food and feed is an essential aspect of human activity; however, it is also one of the most demanding and inefficient. Food waste occurs even at the production stage [12]. The majority of food waste is generated by the consumers. This might be caused by low consumer awareness and knowledge about food storage and processing (both preliminary and thermal processing). The scale of waste is significant enough that when estimating the amount of food required to feed the global population in 2050, a certain level of overproduction must be factored in. Some sources indicate that food production may need to increase by as much as 56%, despite the current global surplus of food products [13]. Such intensive food production, particularly of high-quality animal protein, will also entail a significant environmental cost. This will include high greenhouse gas emissions, the substantial consumption of water, and vast areas dedicated solely to livestock farming and feed production [14].

Another challenge is the continuously rising cost of animal feed, particularly those based on proteins such as fishmeal and soybean meal. Data show that not only are edible insects a cost-effective form of feed but they can also contain more essential amino acids than fishmeal and soybean meal (Table 1). The contributing factors include climate change, prolonged droughts, greenhouse gas emissions, water and land pollution, and high global demand for proteins [15]. The production costs of fish and soy are considered primary indicators of the high feed prices for poultry and pigs, among others [16]. Additionally, the production of edible insects is recognized as low-emission, especially when compared to livestock production [17,18]. Considering all of the above, edible insects are proposed as a viable alternative.

### 2.2. The Economics of Edible Insect Production

It is estimated that edible insects are currently consumed regularly by over 2 billion people in Africa, Asia, and Latin America [19]. People across the world consume over 2000 insect species, with the most popular being beetles, caterpillars, bees, wasps, ants, grasshoppers, locusts, crickets, cicadas, leaf and plant hoppers, scale insects and true bugs, termites, dragonflies, and flies [20]. Published data reports that the farming of edible insects is significantly less costly than livestock farming. In highly developed countries, production takes place in specialized agricultural facilities. However, it is noted that edible insect production could also present a substantial opportunity for developing countries, particularly for lower-income populations in both rural and urban areas [7]. In developing countries, a significant majority of insect harvesting is done in the wild, in forests, or on farms. This solution does not require substantial additional resources to start such activities. Harvesting edible insects could diversify household income. Farmers often use edible insects to feed their own families, though it is noted that any surplus could be sold or exchanged in barter [7]. An additional advantage of this form of trade is that it bypasses intermediaries, allowing those performing the work to retain the maximum share of the income [7].

Edible insect farming, compared to livestock farming, is highly efficient in the use of various resources: water, feed, and space [21,22]. The land surface required for farming and feed production is a crucial factor, as the FAO estimates that arable land available for sustainable use is decreasing. By the year 2050, there shall be a predicted discrepancy of over 100 million hectares between “business as usual” and “sustainable levels” [23]. Additionally, it is noted that livestock production meets only 15% of the global dietary energy demand yet requires approximately 80% of agricultural land, accounting for 3.9 billion hectares [18]. Over the past decade, the European Union has intensified the promotion of edible insects as a protein source for both humans and animals, significantly increasing interest in insect farming and highlighting its numerous benefits [24]. The European Union has intensified its efforts over the past decade to promote edible insects as a source of high-quality protein for both humans and animals. This has significantly increased interest in insect farming and highlighted numerous benefits [15]. However, it is important to emphasize that the production of edible insects in the European Union remains significantly limited, both in terms of the number of approved species and the techniques that can be employed [15,25].

A significant advantage of edible insect farming is their low nutritional requirements, as many species can be fed organic waste. This allows for the transformation of low-value waste into high-quality, valuable protein [15,25]. Another valuable aspect is that up to 80% of the biomass of edible insects can be digested, in contrast to poultry and pork (55%) and beef (40%) [23]. Edible insects also require significantly less water compared to the farming of cattle, pigs, or poultry [26]. The production of 1 g of protein derived from beef and poultry requires, respectively, 500% and 50% more water than mealworm production. Similarly, beef and poultry protein production require 8–14 times and 2–3 times more land than mealworm production, respectively [26,27].

### 2.3. Nutritional Value

One of the main advantages of edible insect production would be to support animal production as feed and also to reduce animal production in general. This could be considered substituting insect proteins for animal proteins. Protein-energy malnutrition (PEM) is considered a major health concern in many regions, especially sub-Saharan Africa (SSA). The WHO estimates that 60% of all deaths in children (under the age of five) can be attributed to malnutrition [28]. The main two causes of malnutrition are PEM and energy deficiency, in varying proportions [29].

#### 2.3.1. Proteins

Insect proteins contain an abundance of every essential amino acid [30] (Table 1). This is the reason why they can be considered a substitute for animal proteins [21]. In addition, edible insects might contain even more proteins than animals, which is essential for developing countries [18]. Based on dry matter, edible insects might contain up to over 60% of proteins [16]. Currently, there are four species that are permitted for consumption by humans in the EU. The aforementioned edible insects have a higher essential amino acid content than FAO/WHO (2007) standards [31]. The amino acid composition of edible insects seems very desirable for feed, especially when compared to fishmeal and soy meal (Table 1).

Plant proteins lack essential amino acids; therefore, it is advisable to enrich plant-based products with animal-based products. It will most likely be beneficiary to combine plant-based flour with edible insect-based flour, as this blend will provide a complete profile of essential amino acids. This integration ensures a balanced source of protein, supporting a more comprehensive nutritional intake than plant-based flour alone. In this manner, edible insects might have a similar role to animal-based protein sources. Incorporating edible insect flour into traditional, plant-based flour is highly advised as it might be a solution to incorporate a valuable protein source while avoiding consumers’ resentment towards the product [32].

**Table 1:** Amino acid composition (g/100 g of protein) and nutritional parameters of edible insects allowed for consumption by humans in the European Union.

Amino Acid	ACD	ALD	LM	TM	Fishmeal	Soy meal
Isoleucine	4.46	4.77	4.66	4.94	2.92	3.01
Leucine	7.79	7.36	8.44	7.88	4.59	4.10
Arginine	7.78	5.91	6.20	5.94	2.33	0.26
Valine	6.59	6.40	7.10	7.13	3.61	2.83
Methionine	2.20	1.71	1.42	1.75	0.97	0.59
Lysine	5.20	7.35	5.01	5.20	6.45	5.96
Histidine	2.92	3.21	2.87	3.55	3.17	2.89
Threonine	4.07	4.42	3.82	4.28	1.41	1.46
Phenylalanine	4.07	4.22	3.23	4.39	6.09	5.51
Total EAAs	45.01	46.44	43.06	48.37	31.54	26.61
Limiting AAs	Lys	Leu	Lys	Lys	Arg	Arg

ACD: *A. domesticus*; ALD: *A. diaperinus*; LM: *L. migratoria*; TM: *T. molitor*; AA: amino acid; EAA: essential amino acid; based on [31,33,34].

A study has shown that wheat, sorghum, and insect-based cookies, as well as being energy-dense, shelf-stable, popular, and ready-to-eat baked goods, are rich in protein and attractive to consumers (both children and adults) [30]. Research also revealed that substituting wheat flour with higher levels of sorghum and insect-based flour led to both a higher protein content and its in vitro digestibility (which increased by 23.8% with the increase in the sorghum–termite blend). Utilizing such a composition and product is in fact very promising, especially due to the very high essential amino acid content in edible insect powders, as shown in Table 1. The combination of sorghum with insect proteins offers a novel and effective approach to improving the nutritional value of food products, particularly in regions where both sorghum and insects are readily available and culturally accepted. This strategy holds promise for combating malnutrition and enhancing food security in vulnerable populations [30].

The functional properties of edible insect proteins have become a subject of interest among researchers. Kim et al. assessed the feasibility of using *Tenebrio molitor*, *Allomyrina dichotoma*, and *Protaetia brevitarsis seulensis* as alternatives to meat proteins [35]. Among these species, *T. molitor* exhibited the best emulsion properties. Emulsions containing mealworm proteins displayed the highest values for hardness, gumminess, chewiness, and apparent viscosity. Among the evaluated insect species, yellow mealworm protein exhibits superior emulsifying properties. The results highlighted the promising potential of mealworm protein. Insect proteins demonstrated higher interfacial activity and faster adsorption kinetics compared to whey proteins, suggesting that emulsions stabilized with insect proteins could be more stable than those with whey [35]. Zielińska et al. concluded that insect proteins offer excellent emulsion stability, supporting their suitability for creating innovative food formulations [36]. Other functional properties of edible insect proteins will be examined in Section 3.2.5.

#### 2.3.2. Fat

Edible insects can contain a substantial amount of fat; however, this might vary greatly depending on the species and their form. The larval form has been reported as the one with the highest fat content. Perez-Santaescolastica et al. examined seven insects, four of which are allowed for human consumption in the European Union. The highest total lipid content was found in *G. mellonella* at 53.63 g/100 g DM (dry matter), while *Z. morio* and *L. migratoria* contained 33.97 and 32.28 g/100 g DM, respectively. Research established that *T. Molitor*, *A. diaperinus*, and *A. Domesticus* contained 22.61, 21.82, and 21.32 g/100 g DM, respectively, and *B. dubia* was reported to have the least fat at 13.96 g/100 g DM) [31]. These values correspond with results reported by other authors [37,38,39,40], with the exception of TM, which had lower values than those reported by [40]. ZM, TM, ALD, and GM were obtained in larval form, whereas BD, ACD, and LM were collected in their adult stages. This corresponds with the requirements of Commission Implementing Regulation (EU) 2017/2470 of 20 December 2017, which establishes the list of novel foods in accordance with Regulation (EU) 2015/2283 of the European Parliament and of the Council. According to this, ACD and LM are allowed in their adult forms, whereas only the larvae of ZM and ALD are admissible. The data presented in Table 2 highlight that edible insects, unlike meat, contain a lot of unsaturated fatty acids, both mono and poly.

#### 2.3.3. Carbohydrates

In general, edible insects are not considered to be a rich source of carbohydrates. The most prominent fraction of carbohydrates is fiber, which consists mostly of chitin [27]. It is an indigestible, main component of arthropod exoskeletons, tendons, and the linings of their respiratory, excretory, and digestive systems [41]. Despite chitinase being present in the human gastric tract, this enzyme is reported to be only active in people who are accustomed to eating insects [14]. Chemically, chitin consists of insoluble polysaccharides made up of N-acetylglucosamine monomers bound together with *β*-(1–4)-N-acetyl-dglucosamine bonds. It also contains around 6–7% nitrogen, which ought to be retracted from the overall nitrogen content [27,41,42]. Besides chitin, glycogen was reported as one of the carbohydrates in edible insects [43].

Chitin is one of the most abundant renewable biopolymers on earth and can be obtained as a cheap renewable biopolymer. It is biocompatible, biodegradable, and bio-absorbable, with antibacterial and wound-healing abilities and low immunogenicity. Therefore, there have been many reports on its biomedical applications. Furthermore, it is also utilized in different fields, such as food technology, materials science, microbiology, agriculture, wastewater treatment, drug delivery systems, tissue engineering, and bionanotechnology [41].

Nonetheless, chitin might also compromise the bioavailability and digestibility of proteins and other substances. The adverse effects of consuming chitin derived from edible insects will be characterized in Section 3.

#### 2.3.4. Minerals

Edible insects have a high content of essential minerals, such as calcium (Ca), magnesium (Mg), iron (Fe), and zinc (Zn) [44]. Edible insects are particularly valued for their ability to provide macro and trace minerals essential for human health, including Mg, Fe, Mn, and Zn. Studies show that the mineral content can vary significantly depending on the species, life stage, and feeding substrate. For instance, *Tenebrio molitor* larvae, when reared on wheat bran supplemented with carrots and potatoes, exhibited exceptionally high mineral concentrations, reaching up to 335,105 mg/100 g of dry matter [44,45]. Similarly, wild-harvested insects, such as *Onyoso mammon* in Kenya, demonstrated an elevated Fe content, which is crucial for combating iron-deficiency anemia, particularly in developing regions [46].

The diet and environmental conditions under which edible insects are cultivated significantly influence their mineral profiles. For example, the silkworm *Samia ricinii* displayed variations in its mineral content when fed castor leaves versus tapioca leaves, indicating that feeding substrates play a pivotal role in determining nutrient composition [47]. Furthermore, geographical differences impact mineral accumulation, as observed in *Rhynchophorus phoenicis* larvae from Angola and Nigeria, which exhibited notable variations in Ca, Fe, and P concentrations [48]. Such differences underline the need for standardized rearing practices to ensure consistency in nutrient content.

Despite their nutritional benefits, edible insects can accumulate nonessential and potentially hazardous elements, such as cadmium (Cd), lead (Pb), and mercury (Hg), from their environment, especially when wild-harvested or reared in contaminated conditions [49,50]. However, studies on reared insects such as *Tenebrio molitor* and *Zophobas morio* have shown that heavy metal levels remain below regulatory safety limits, rendering these insects safe for human consumption [51]. Nevertheless, the risk of contamination in wild species persists, as insects can absorb heavy metals through contaminated plants, soil, or water sources [44].

The pathways through which minerals are incorporated into insects primarily involve their diet, including natural feed or artificial substrates. While bioavailability studies have demonstrated that minerals such as Fe and Zn are highly absorbable in edible insects, processing methods can significantly affect nutrient retention. For instance, boiling has been shown to reduce the bioavailability of Fe and Zn in certain grasshoppers, whereas roasting preserves mineral content more effectively [43]. Additionally, heavy metals consumed by insects may be sequestered in the digestive system or excreted through feces, reducing their potential toxicity [44].

#### 2.3.5. Vitamins

The vitamin composition of insects varies depending on the species, developmental stage, and diet, but overall, insects offer notable quantities of water-soluble and lipophilic vitamins that contribute to their nutritional value. Among the B-complex vitamins, thiamine (B1), riboflavin (B2), and vitamin B12 have been identified in significant amounts. For instance, the thiamine content in edible insects ranges from 0.1 to 4 mg per 100 g of dry matter, while riboflavin concentrations span 0.11 to 8.9 mg per 100 g, depending on the species analyzed [14].

Particularly noteworthy is the high vitamin B12 content observed in certain insect species. The larvae of the yellow mealworm beetle (*Tenebrio molitor*) contain 0.47 μg per 100 g, whereas the house cricket (*Acheta domesticus*) demonstrates even higher concentrations, with 5.4 μg per 100 g in adults and up to 8.7 μg per 100 g in nymphs [14]. This is especially valuable in addressing vitamin B12 deficiencies that are common in populations relying on plant-based diets, as this vitamin is predominantly found in animal-derived foods.

Insects also contain varying levels of fat-soluble vitamins, particularly vitamins A, D, and E. For example, retinol (preformed vitamin A) and β-carotene were detected in some caterpillars, such as *Imbrasia oyemensis*, *Nudaurelia oyemensis*, and *Ichthyodes truncata*. These species provide 32–48 μg of retinol and 6.8–8.2 μg of β-carotene per 100 g of dry matter. In contrast, commonly farmed species such as T. *molitor* and A. *domesticus* contain only trace amounts of these vitamins, suggesting that the vitamin A content is highly species-specific [14,52].

Vitamin E, particularly α-tocopherol, is another essential nutrient found in edible insects. Red palm weevil larvae (*Rhynchophorus ferrugineus*) are notable for their high α-tocopherol content, averaging 35 mg per 100 g of dry matter, with additional tocopherols such as β- and γ-tocopherol contributing 9 mg per 100 g. Similarly, silkworms (*Bombyx mori*) contain 9.65 mg of tocopherols per 100 g, making these species a significant source of vitamin E [53].

On the other hand, vitamin C and niacin (B3) are present in minimal quantities or are absent in many edible insect species. This variability underscores the need for a comprehensive analysis of vitamin content across different species and rearing conditions. Moreover, the feed provided to farmed insects can be manipulated to enhance their nutrient profiles, including vitamin content, making edible insects a versatile food source adaptable to specific nutritional requirements [39].

While edible insects are generally not a major source of vitamin A or C, their richness in B-complex vitamins and vitamin E highlights their potential as a functional food, particularly in addressing micronutrient deficiencies. Further research is required to standardize the vitamin profiles of farmed insects and explore the bioavailability of these nutrients in the human diet. With appropriate processing and dietary integration, edible insects offer a sustainable and nutrient-rich alternative to traditional food and feed sources (Table 3) [39].

## 3. Challenges of Popularizing Edible Insects

When considering the benefits of insect production and consumption, it is impossible to overlook the associated risks. Insect consumption encounters consumer reluctance, insufficient comprehensive research on microbiological safety and its impact on human health, and chemical safety during production and processing, as well as potential fraud throughout the supply chain. Information is also scarce with respect to certain environmental threats. In the context of the risks and challenges that may arise from the production and consumption of insects, several areas can be distinguished: awareness and cultural aspects, technical and technological aspects, and health safety for humans and animals, as well as environmental safety, which is primarily focused on the natural environment, and labeling, understood as ensuring the informational safety of the product.

### 3.1. Consumer-Based Challenges

Disgust and the perception of insects as pests are the most common reasons for rejecting them. Food neophobia plays a key role in shaping the acceptance of edible insects by Western societies [63]. Research indicates that neophobic attitudes are more frequently exhibited by individuals who prefer meat. However, if insects were served in a different form, such as flour added to baked goods, acceptance of such a product increased [64]. Additionally, there are strategies that try to mitigate disgust towards edible insects and neophobia, including tasting sessions or cooking shows, among others [65].

### 3.2. Non-Consumer-Based Challenges

#### 3.2.1. Inedible Insect Parts

One of the factors limiting the consumption of insects is the presence of sharp spines on insect legs. Mlcek et al. report that as early as 1945, Bouvier observed in the Democratic Republic of Congo that consuming whole locusts and grasshoppers could lead to intestinal problems caused by the spines on the insects’ legs. Autopsies of monkeys that died during locust invasions also revealed that consuming locusts resulted in their death due to the same reason [66].

One of the reasons that may limit the consumption of insects is the presence of antinutritional compounds. Among these, chitin is the most often mentioned due to its potentially adverse effect on protein digestion [67] and is itself considered indigestible. Although, the latter finding might not be entirely true as there have been reports of human chitinases, such as chitotriosidase 1 (CHIT1) and acid mammalian chitinase (AMCase), along with several chitinase-like proteins (CLPs). However, their role has been mostly investigated in relation to their protective role against pathogens through chitin degradation. Mammalian chitinases are now gaining attention as the key players in innate immune responses against fungi, bacteria, and other pathogens [68]. Additionally, recently discovered chitinolytic enzymes produced by bacteria in the human gastrointestinal tract suggest that chitin and chitosan may be digestible [38]. Research by Refael et al. (2022) suggests that insect-derived chitin could potentially be a new prebiotic, though further studies are needed to confirm this concept [69].

Chitin and chitosan possess significant potential, which can be utilized in food and nutrition, as well as in the pharmaceutical, cosmetic, and dietary supplement industries [70]. These compounds may have a wide range of biomedical applications, including wound healing, tissue engineering, drug delivery, and antimicrobial therapies. Their antimicrobial properties open up possibilities for innovative solutions in various medical interventions [71]. Chitosans have also been successfully implemented in food packaging for years (Tripathi chitosan films). Waste generated from the farming and processing of edible insects should be collected and considered as an alternative source of chitin/chitosan [72]. Properly prepared insects do not pose a threat to consumers.

#### 3.2.2. Antinutrients and Allergenicity

Other antinutritional compounds present in insects include tannins, phytates, oxalates, and cyanogenic glycosides. These compounds disrupt mineral balance and chelate proteins, with oxalates additionally impairing kidney function [73,74]. A particular case of an antinutritional effect is the enzyme thiaminase, which is responsible for the seasonal ataxia observed after consuming the roasted larvae of *Anaphe venata* by the population of Nigeria [75].

Another concern raised by the scientific community is the allergenicity of insects. Certain types of proteins present in edible insects, including arginine kinase, are considered allergens [76]. Insects are closely related to crustaceans, which suggests that they might trigger food allergies [77]. Cases described in the literature do not confirm widespread allergic reactions among insect consumers. However, insects should be consumed cautiously, especially when being introduced into the diet for the first time. Further research is needed to assess the risks associated with food allergies to edible insects [78]. Furthermore, according to Commission Implementing Regulation (EU) 2017/2470 of 20 December 2017, which establishes the Union’s list of novel foods under Regulation (EU) 2015/2283 of the European Parliament and Council on novel foods, food products containing edible insects must include a statement indicating that this ingredient may cause allergic reactions in consumers with known allergies to crustaceans, mollusks, and related products, as well as to mites.

At this point, it is worth revisiting the previously discussed risk associated with insects, namely chitin. While chitin is not widely regarded as a potential allergen, it can cause sensitization through frequent exposure [79]. This risk could affect, for example, workers on insect farms. Such allergies have been reported previously by Schroeckenstein et al. (1990), who noted that beetles from the *Tenebrionid* family are potentially significant allergens for workers exposed to grains or grain products [80].

#### 3.2.3. Biological Risks

Among biological risks, pathogenic microorganisms and parasites are highlighted. Pathogenic bacteria such as *Escherichia*, *Staphylococcus*, and *Bacillus* can infect both humans and insects [76]. Edible insects serve as hosts for potentially dangerous bacterial species, including *Campylobacter*, *Bacillus*, *Staphylococcus*, *Neisseria*, *Pseudomonas*, and *Clostridium*. These insects can contribute to foodborne diseases. Having considered collecting insects from natural environments in developing countries, primarily in Africa and Asia, ensuring food safety will be particularly challenging [75,81].

Entomophagy can facilitate the transmission of parasites from insects to humans. *Dicrocoelium dendriticum* is a zoonotic parasite that can be easily transmitted to humans through the consumption of edible insects, such as ants. Parasites such as *Entamoeba histolytica*, *Giardia lamblia*, and *Toxoplasma* spp. have been isolated from cockroaches [82]. For this reason, consuming raw insects is not recommended, and they should undergo appropriate processing. Common methods for preserving insects include reducing the water content (drying or freeze-drying), acidification, or thermal processing (boiling, blanching, or sterilization) [83].

#### 3.2.4. Chemical Risks

Chemical contamination in insects includes pesticides, heavy metals, and mycotoxins. Pesticide residues pose a particular risk for insects collected from the wild. Collectors often lack awareness or disregard whether the agricultural fields where insects are found have been treated with pesticides. The literature provides limited data on pesticide residues in insects, primarily concerning Asian countries [84]. The *Codex Alimentarius* recommends that the concentrations of chlorpyrifos and piperonyl butoxide in insect feed, such as alfalfa and field peas, be lower than the permissible levels for livestock feed, specifically 5000 μg/kg and 2000 μg/kg, respectively [85].

Heavy metals such as cadmium, lead, mercury, and arsenic accumulate in insects, with the extent of accumulation depending on the specific metal, insect species, and growth stage [86]. The EFSA reports that heavy metals such as cadmium and arsenic can accumulate in edible insects when they are fed contaminated feed or inhabit polluted substrates [85]. However, research by Poma et al. (2017) conducted in Belgium found that insects and products derived from them contained fewer metal contaminants than other commonly consumed animal products. This is particularly true for farmed insects, as insect farms allow for proper monitoring and control [87].

Mycotoxins are secondary metabolites produced by various phytopathogenic molds, including species of *Fusarium*, *Aspergillus*, and *Penicillium*. They are significant food contaminants with acute and chronic adverse effects on human health. Mycotoxins may originate from contaminated feed or substrates used for rearing edible insects [82,88].

The EFSA (2015) highlights that other chemical substances may be used during insect farming, such as biocides for cleaning facilities and equipment or veterinary drugs for treating certain diseases [85]. Moreover, some insect species naturally produce toxins (venoms). Furthermore, EU regulations state that a minimum 24 h fasting period is required to allow the larvae and adult forms to discard their bowel content before killing and processing the insects [89]. We do not know the hazards if the 24 h fasting period is not applied.

#### 3.2.5. Functional Properties of Edible Insect Proteins

Edible insect proteins are deemed to be an animal protein alternative. To properly evaluate its potential to be utilized as such, their functional properties have to be thoroughly investigated. There are several factors that should be taken into consideration: solubility, foaming, and emulsification.

High solubility can be an indicator that the protein is highly digestible, which makes it a desirable trait for protein application [90]. Studies report that some edible insects have the lowest solubility at pH 4–5 and the highest at 10–11 for protein preparations obtained via combined alkaline extraction and isoelectric precipitation [36,91,92]. However, the solubility differs depending on the processing method. The use of a fluidized bed, microwave, and rack oven for drying yellow mealworms reduced protein solubility to just 12.65–19.25%, in contrast to freeze-dried (40.65%), vacuum-dried (49.70%), and fresh (53.24%) mealworms [93,94].

Yi et al. investigated the foaming and gelling properties of proteins extracted from five insect species: *Tenebrio molitor*, *Zophobas morio*, *Alphitobius diaperinus*, *Acheta domesticus*, and *Blaptica dubia*. Their performance was compared to that of chicken egg white albumin. At pH levels of 3, 5, 7, and 10, the insect proteins exhibited significantly poorer results than albumin. Moreover, when foams were produced, they were found to be unstable [95].

Edible insect proteins exhibit significant gelling properties, which are valuable for food applications such as jellies, desserts, yogurts, and meat products. The ability of these proteins to form gel structures depends on factors such as pH, temperature, protein concentration, and the presence of salts [9]. Research has shown that proteins extracted from various insect species, such as *Tenebrio molitor* and *Acheta domesticus*, gel within a pH range of 7–10 at high protein concentrations (approximately 30% *w*/*v*). The gelation temperature for *Tenebrio molitor* proteins was found to be around 61.7 °C, while for *Acheta domesticus*, it was approximately 56.2 °C [9,95].

External factors, including the addition of salt (e.g., NaCl), pH alterations, and controlled thermal treatments, significantly influence the gelation process. Higher protein concentrations and specific thermal conditions promote the transition from sol to gel, resulting in the formation of a three-dimensional network. These properties are also influenced by the protein profile, which varies depending on the life stage of the insects. Proteins extracted from adult insects generally exhibit stronger gel-forming abilities compared to those from larvae. Such differences are attributed to the intrinsic structural characteristics of the proteins that determine their functionality [9,96].

These findings highlight the potential of insect proteins as gelling agents, offering a sustainable and functional alternative to conventional ingredients in food formulations.

#### 3.2.6. Insect Populations Worldwide

In the research on the impact of insects on the environment and the influence of the environment on insects, two main directions can be observed. The first focuses on the declining number of insects worldwide, while the second highlights the risks associated with industrial insect farming, including the potential release of insects into the environment due to various factors. Van Huis and Oonincx (2017) emphasize the threats to aquatic insect populations caused by water pollution, the disappearance of caterpillar species in Africa due to excessive deforestation, which destroys their habitats, and the decline of edible insect species regarded as pests and eradicated in agroecosystems [18].

#### 3.2.7. Ethical Aspects of Rearing and Consuming Edible Insects

The ethical discourse surrounding edible insect farming centers on animal welfare, sentience, and precautionary principles. While insects are increasingly considered a sustainable protein source, their inclusion in animal welfare legislation remains inconsistent and often insufficient. Scientific evidence suggests insects possess nociceptive capacities and may exhibit pain-like responses, though sentience remains unconfirmed [97]. However, due to epistemological limits in assessing consciousness, it might be crucial for precautionary ethical treatment. This includes minimizing harm during rearing and using humane slaughter methods (e.g., freezing over boiling).

Sentience, particularly the capacity to experience aversive states, should be viewed as a threshold for moral consideration. Though insect neuroanatomy differs from vertebrates, emerging research indicates possible analogs to pain processing systems. Ethically, if insect sentience is probable, their welfare merits protection, especially given the high numbers used in mass farming [97].

Pragmatically, farming insects may reduce overall animal suffering compared to conventional livestock. Nonetheless, significant knowledge gaps remain regarding species-specific welfare needs. Establishing ethical rearing practices and regulatory frameworks is essential before scaling insect farming systems.

## 4. Discussion

Popularizing edible insects is proposed as one of the solutions for feeding a growing human population. Having estimated the global population in 2050, the crucial production of sufficient quantities of food of adequate quality will become extremely challenging. It will be even more demanding in developing countries, as their population growth will be the most dynamic.

In the context of risks associated with insect farming and its impact on the environment, the challenge lies in the fact that conventional food production systems are governed by established legal regulations, whereas knowledge and regulations applicable to commercial insect farming remain fragmented [98]. Consequently, numerous challenges must be addressed during the development of the edible insect market. These challenges pertain to policy formulation, legislative solutions, and production and control standardization, as well as the potential certification of mass-produced edible insects. Welfare standards for each species of farmed insect are essential. Additionally, logistical operations cannot be effectively executed without the implementation of appropriate regulations and procedures. At present, edible insects fall outside the scope of veterinary regulations that ensure the safety of animal production within the European Union [98].

Another issue requiring research is the concern over the potential adulteration of products containing insects, whether whole, ground, or in the form of isolated proteins. Food fraud is a global problem of increasing significance that can harm both human and animal health. The globalization of food supply chains offers many benefits in terms of food variety and availability but can also increase the risk of fraud. The lack of regulations and standards regarding the authenticity of insects and insect-derived products will hinder efforts to combat illegal activities within supply chains [99].

The benefits of utilizing insects as food and feed are undeniable (Table 3), although there are numerous challenges. Additionally, the continuous decline in insect populations receives limited attention across various domains, from scientific research to policymaking and conservation efforts. Scientists have urgently called for prioritizing insect conservation. It also highlights that global treaties on pollinator care and the restoration of pollination ecosystems are urgently needed [100].

## 5. Conclusions

The main advantages of insect farming include lower land and water requirements, reduced greenhouse gas emissions, high feed conversion efficiency (i.e., weight gain relative to feed intake), and the ability to transform low-value organic by-products into high-quality food or feed.

There are numerous items of research and reports referring to the many benefits of utilizing edible insects as a sustainable food source. Rearing edible insects is much more efficient than livestock production, especially when considering greenhouse gas emissions, feed conversion ratios, areas needed for production, and the generally low cost of production. Additionally, edible insects might be considered a method of converting organic waste into high-quality proteins.

Current EU regulations permit only four species of insects to be used as food. Additionally, there are only certain forms and stages of development that are allowed to be utilized. It highlights how problematic and time-consuming adaptation can be. Despite the slow pace of adoption, neophobia and general disgust towards insects are still relevant and will probably remain relevant for years to come. There is also one additional condition that is opposed to sustainable development. Should humanity decide to farm only a few species on a mass scale, it will most definitely stand against biodiversity.

Having considered the aforementioned arguments, it would be highly advisable to revisit the global stance on edible insects as a sustainable food source. There are still too many variables that have not been fully investigated. While edible insects are definitely nutritious and relatively easy to produce, each species and stage of development might be significantly different. Therefore, it is essential to conduct further research on edible insects to collect the required data.

## Figures and Tables

**Table 2:** The fatty acid profiles and the total lipid content of edible insects, expressed as a percentage.

Parameter	ACD	ALD	LM	TM
SFA	35.18 ± 0.45	36.41 ± 0.98	39.21 ± 0.44	31.17 ± 2.94
MUFA	25.06 ± 0.05	39.21 ± 0.47	33.02 ± 0.08	38.42 ± 0.67
PUFA	39.76	24,38	27.77	30.41
PUFA/SFA	1.13	0.67	0.71	0.98
n-3	2.75	1.15	10.67	1.49
n-6/n-3	13.47	20.16	1.59	19.43

ACD: *A. domesticus*; ALD: *A. diaperinus*; LM: *L. migratoria*; TM: *T. molitor*; SFA: saturated fatty acid; MUFA: monounsaturated fatty acid; PUFA: polyunsaturated fatty acid; based on: [31].

**Table 3:** Characterization of different protein types.

Aspect	Edible Insects	Livestock Animals	High-Protein Plants
Protein content (fresh matter)	10–25%	poultry 18–24%, beef 20–24%, pork 9–22%	38–46.5% soy
Feed conversion ratio (FCR)	~2:1	8–10:1 (cattle), 3–4:1 (pigs), 2:1 (poultry)	does not apply
Production time	4–8 weeks	6–36 months	3–6 months (seasonal)
Water usage (per 1 kg protein)	4–6 L	112–15,000 L	500–1800 L
CO_2_ emissions (per 1 kg protein)	<1 kg	14–30 kg (cattle), 3–5 kg (poultry)	0.5–2 kg
Land use	~15–20 m^2^/ton	~200–250 m^2^/ton	~50–100 m^2^/ton
Waste/by-products	biofertilizer, low waste	large amount of waste	often edible or usable as feed
Production costs	low with scale and automation	high (feed, energy, veterinary)	dependent on yield and season
Consumer acceptance	low–moderate (Europe), increasing	high	high and increasing (vegetarianism, flexitarianism)
Urban farming potential	very high	very low	limited (fields)

Based on [8,34,54,55,56,57,58,59,60,61,62].

## Data Availability

No new data were created or analyzed in this study. Data sharing is not applicable to this article.
